# Toward human-resolution haptics: A high-bandwidth, high-density, wearable tactile display

**DOI:** 10.1126/sciadv.adz5937

**Published:** 2025-11-19

**Authors:** Sylvia Tan, Michael A. Peskhin, Roberta L. Klatzky, J. Edward Colgate

**Affiliations:** ^1^ Department of Mechanical Engineering, Northwestern University, Evanston, IL 60208, USA.; ^2^ Department of Psychology, Carnegie Mellon University, Pittsburgh, PA 15213, USA.

## Abstract

Despite advances in digitizing vision and hearing, touch still lacks an equivalent digital interface matching the fidelity of human perception. This gap limits the quality of digital tactile information and the realism of virtual experiences. Here, we introduce a step toward human-resolution haptics: a class of wearable tactile displays designed to match the spatial and temporal acuity of the human fingertip. Our device, VoxeLite, is a 0.1-millimeter-thick, 0.19-gram, skin-conformal array of individually addressable soft electroadhesive actuators (“nodes”). As users touch and move across surfaces, VoxeLite delivers high-resolution distributed forces via the nodes. Enabled by scalable microfabrication techniques, the display achieves actuator densities up to 110 nodes per square centimeter, produces stimuli up to 800 hertz, and remains transparent to real-world tactile input. We demonstrate its ability to render small-scale hapticons and virtual textures and transmit physical surfaces, validated through human psychophysics and biomimetic sensing. These findings position VoxeLite as a platform for human-resolution haptics in immersive interfaces, robotics, and digital touch communication.

## Teaser

Human-resolution haptic display uses skin-conformal actuators to deliver lifelike touch for virtual and real-world interactions.

## INTRODUCTION

While we can readily digitize what we see and hear, the same has not yet been achieved for touch. Liquid crystal displays can produce hyperrealistic images (*1*), and miniature dynamic transducers enable immersive spatial audio (*2*). Physical touch, however, still lacks a true digital counterpart. Despite its central role in how we perceive, navigate, and connect with the world (*3*–*5*), we lack a meaningful way to create realistic digital touch.

Today’s most common form of haptic feedback is vibration, useful for alerts but incapable of conveying the richness and nuance of real tactile experiences. Unlike sight and sound, which can be digitized into pixels and waveforms, touch involves complex patterns in both space and time. It also requires the transfer of physical force into the body, rather than pure electronic simulation. Creating these sensations, especially at the fingertips, is particularly challenging. Human fingers have a spatial acuity of ~1 mm and are sensitive to a wide frequency range of 0 to 1000 Hz (*6*–*8*). Any tactile display that seeks to recreate realistic touch experiences must meet these human-resolution performance standards while remaining lightweight and mechanically unobtrusive.

Previous studies have explored temporally modulated vibrations alone to convey fine tactile information (*9*–*13*), but even faithful signal replay failed to produce realistic textures (*14*). Incorporating spatially distributed actuation has improved performance in rendering tactile graphics and texture. These devices, however, suffer from a low temporal resolution (*15*–*18*) and/or rigid, bulky form factors that are incompatible with naturalistic, wearable use (*16*–*19*). Notably, works by Grigorii *et al.* (*20*) and Massalim *et al.* (*21*) underscore the importance of combining fine spatial resolution with high temporal fidelity: Millimeter-scale spatial features crucially influence perception, even at relatively high frequencies such as 80 Hz. However, even systems with 2-mm actuator spacings have not achieved perceptually realistic textures (*17*), pointing to a need for human-resolution level actuation.

**Fig. 1.**
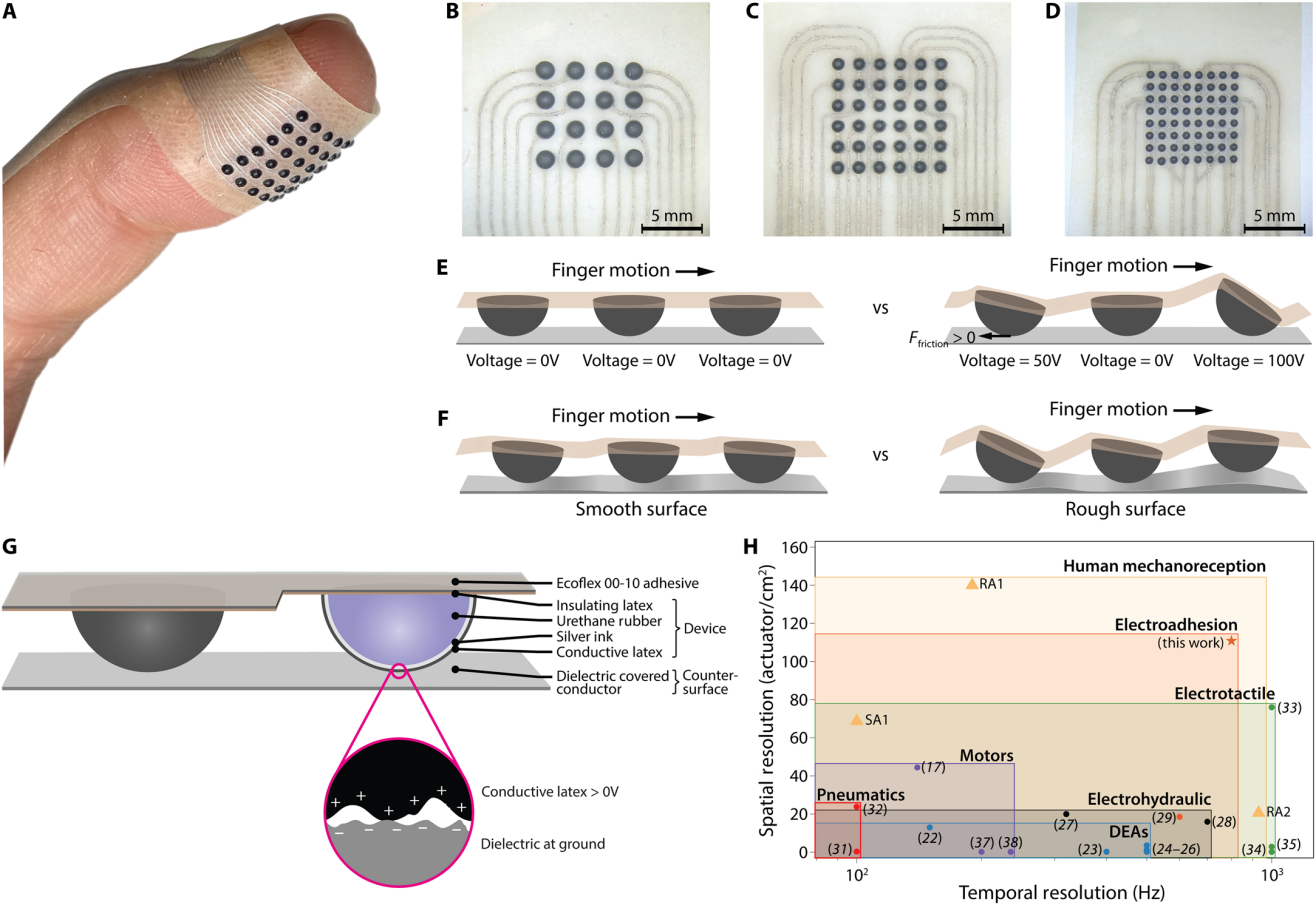
Design and working principle of VoxeLite. (**A**) A 6-by-6 haptic array conformed to the finger and provides high spatiotemporal tactile information within the fingerpad. (**B**) A 4-by-4 version, each node 1.5 mm in diameter, spaced 2.5 mm center to center; (**C**) a 6-by-6 version, each node 1 mm in diameter, spaced 1.6 mm center to center; (**D**) an 8-by-8 version, each node 0.635 mm in diameter and spaced 1 mm center to center. (**E** and **F**) Illustrations of the working mechanism of VoxeLite in active (E) and passive (F) states. In an active state, high voltages generate frictional forces that cause the node to tilt and indent into the skin. In a passive state, the nodes move in response to the micro- and macrogeometries of the object the user touches. (**G**) The layer stacks up of VoxeLite, and all nodes in every array configuration are identical. (**H**) Comparing the space of temporal and spatial resolutions existing wearable tactile devices can achieve, where each box represents the independent maximum spatial and temporal resolutions a given type of actuation can achieve. SA1, RA1, and RA2 are human mechanoreceptors that represent the amount of information the human finger is capable of sensing. DEAs, dielectric elastomer actuators.

The challenge becomes even more formidable when a high spatiotemporal resolution must be achieved in a soft, lightweight, and conformable form factor. To address this, researchers have turned to alternative actuation and fabrication strategies. Dielectric elastomer actuators (*22*–*26*), electrohydraulics (*27*, *28*), electrostatics (*29*, *30*), pneumatics (*31*, *32*), electrotactile (*33*–*35*), and miniaturized motors (*36*–*38*) have shown promise, but none has fully met the combined demands of spatiotemporal resolution and wearability (Fig. 1H).

As a pathway toward human-resolution wearable tactile displays, we present here a class of haptic devices, “VoxeLite.” They are lightweight and skin-conformal, have a high temporal bandwidth, and can generate finely spatially distributed three-dimensional (3D) forces to the finger pad. The devices consist of arrays of independently addressable soft electroadhesive actuators (“nodes”) that conform naturally to the skin, enabling unobstructed interaction with physical surfaces. To support high-resolution tactile rendering, we developed a set of microfabrication techniques that allow for precise and scalable tuning of actuator density, from 20 to 110 nodes/cm^2^, while maintaining mechanical flexibility and minimal weight. Experimental characterization with both a human finger and a biomimetic sensor captured the operating mechanism and capabilities of the device. User studies using a 44 nodes/cm^2^ array further demonstrate its capacity to render fine textures with high perceptual fidelity.

## RESULTS

### VoxeLite design

VoxeLite consists of arrays of individually controlled electroadhesive hemispherical nodes. Custom microfabrication methods enabled the production densities up to 110 nodes/cm^2^, and nodes were patterned in any configuration. Here, we present three versions using square grid patterns: a 4-by-4 array with 1.5-mm-diameter nodes spaced 2.5 mm center to center, a 6-by-6 array with 1-mm-diameter nodes spaced 1.6 mm center to center, and an 8-by-8 array with 0.635-mm-diameter nodes spaced 1 mm center to center (Fig. 1, A to D). Across all configurations, the supporting layers of the device were 0.1 mm thick. Excluding the flexible flat cable, the devices weigh 0.361 ± 0.04 g (4 by 4), 0.186 ± 0.01 g (6 by 6), or 0.3 ± 0.01 g (8 by 8).

**Fig. 2.**
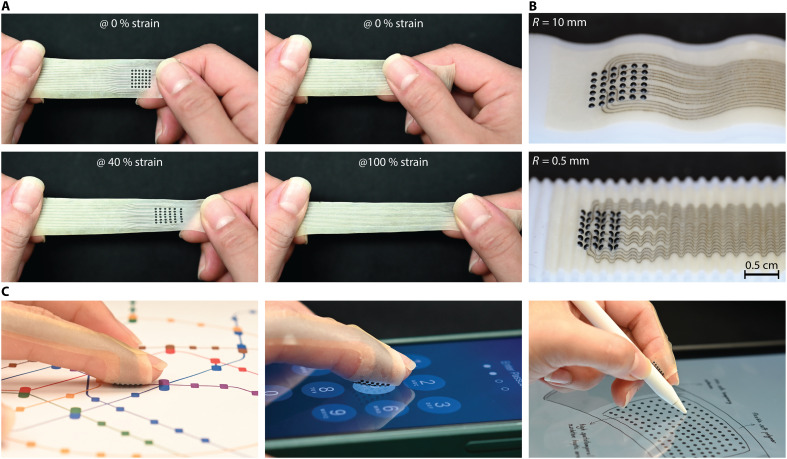
Stretchability, conformability and applications of VoxeLite. (**A**) VoxeLite can be stretched up to 100% strain without an effect on the functionality of the device. At the nodes, thinner tracers are used, which can withstand 40% strain. (**B**) VoxeLite conforming to surfaces made of patterned waves with different radii of curvature, *R*. Radii selected include curvatures found in deformed and undeformed fingers (*45*) (**C**) Example usage of VoxeLite in applications of contour tracing, button localization, and augmented reality interactions.

Each node is composed of four layers: (i) nonconductive latex, (ii) a stiffening rubber, (iii) stretchable silver electrodes, and (iv) a weakly conductive latex (Fig. 1G). The nonconductive latex is an insulating layer between the skin and electrodes, the stiffening rubber provides structure to the node, the stretchable silver electrode transfers high-voltage (60 to 200 V) signals from an external electrical board to the nodes, and the weakly conductive layer interfaces with an electrically grounded countersurface to generate electrostatic forces that increase node-surface friction (see Materials and Methods and fig. S1 for the full fabrication process). The rest of VoxeLite consists of flexible silver electrodes laminated between two nonconductive latex layers. Constructed entirely from soft materials, the device is stretchable and conforms easily to various finger sizes (Fig. 2, A and B).

To wear VoxeLite, the finger is first wrapped with a 50-μm layer of Ecoflex 00-10 silicone rubber, to which the device is attached to such that the flat surface of the hemisphere faces the skin. Ecoflex 00-10 was selected for its tacky surface, which acts as a soft adhesive layer, and for its mechanical properties: It has a significantly lower modulus than skin [800% elongation at break (*39*)], ensuring minimal interference with the node motions.

When not actuated, the nodes passively respond to surface contours, transmitting tactile information directly to the user’s skin (Fig. 1F). This transmission is haptically transparent if the nodes have no intrinsic masking effect, which may depend on the surface texture.

### Principle of actuation

**Fig. 3.**
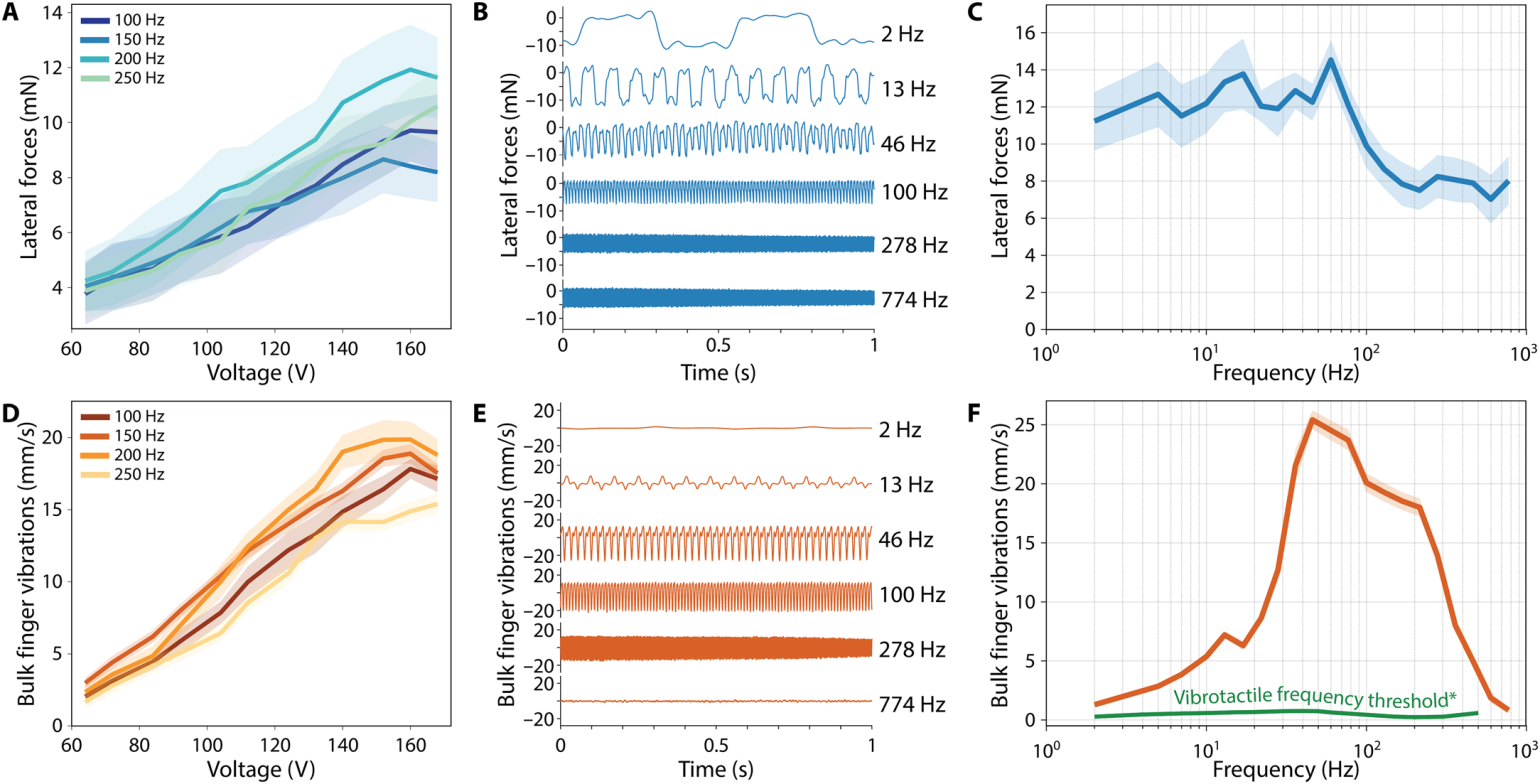
Characterization of the haptic array. (**A**) Relationship between voltage and lateral forces that can be generated across four frequencies. (**B**) Example force response at 2, 13, 46, 100, 278, and 774 Hz. (**C**) Frequency response characteristic relating lateral forces to stimulus frequency. (**D**) Relationship between voltage and bulk finger vibrations experienced across four frequencies. (**E**) Example vibration signals at 2, 13, 46, 100, 278, and 774 Hz. (**F**) Frequency response characteristic relating bulk finger vibrations to stimulus frequency. All vibrations measured are above the vibrotactile frequency threshold of the finger.

VoxeLite operates in two modes: active and passive. In the active state, the user moves their finger across a smooth grounded conductive plate, while a signal is applied to individual nodes. At high voltages (60 to 200 V), electrostatic forces are generated between the outermost layer of the node (weakly conductive latex) and the grounded surface (Eq. 3). When the finger is moving, these increases in electrostatic forces are translated into increased friction forces at the node-plate interface, inducing a tilting motion. As a result, one edge of the node’s flat top indents into the skin (Fig. 1E). Because the actuation is electrically driven, the indentation can be modulated at high temporal frequencies. Users perceive these vibrations from 2 to 800 Hz (Fig. 3, A to F), a bandwidth sufficient to stimulate all four classes of cutaneous fibers, which respond preferentially to different frequency ranges (*8*).

The magnitude of node displacement depends on the frictional force generated at the node-plate interface, which scales with the applied electrostatic force. As voltage increases, both the change in friction and the resulting amplitude of perceived vibration increase proportionally (Eq. 3). This trend holds consistently across frequencies at a given normal preload (Fig. 3, A and D).

The magnitude of electrostatic forces that could be generated was found to depend on the nodes’ mechanical and electrical properties, as well as the amount of normal preload. The mechanical and electrical properties were respectively varied in an initial test by changing the modulus of the stiffening urethane rubber and the concentration of carbon nanotube (CNT) in the conductive latex (see Materials and Methods for more details). Using 2.48 wt % CNT and Shore 40A rubber yielded the highest frictional forces and bulk finger vibrations (figs. S2 and S3). These values were kept constant in all further testing.

The effect of normal preload was also examined (see Materials and Methods for details). Lateral force generation increased with preloads up to 0.8 N, after which it declined. In contrast, the bulk finger vibrations decreased with increasing preload as a result of the increasing apparent stiffness of the finger (fig. S4). As stiffness increased, the node motions were constrained, and vibration transmission was reduced. To balance these trade-offs while ensuring user comfort, a preload of 0.5 N was selected for all subsequent experiments.

In the passive state, no electrical signal is applied. When the user interacts with natural textures and objects, the nodes respond mechanically to variations in the surface friction and geometry. On low-friction surfaces, the nodes produce small indentations, whereas on high-friction surfaces, large indentations are produced. In addition, micro- and macroscale geometries are transmitted through tilting motions as well as whole-node translations (Fig. 1F).

### Characterizing temporal resolution

The temporal resolution of VoxeLite was characterized with a custom-built tribometer, similar to that by Tan *et al.* (fig. S5) (*29*). The device was worn on a real human finger, and the normal force and scan speed were fixed at 0.5 N and 20 mm/s, respectively. The incidence angle of the finger was adjusted such that the array lay parallel to a grounded anodized aluminum countersurface. Bulk finger vibrations were measured using a laser Doppler vibrometer directed at a reflective tape adhered to the side of the finger. Lateral forces were recorded using a piezoelectric force sensor (model 9203, Kistler) mounted to the side of the countersurface.

Initial device calibration was performed via visual inspection with a level to ensure parallel orientation. This was followed by perceptual calibration: The positions of the finger and device were iteratively adjusted on the basis of subjective feedback until uniform sensation was reported across all nodes in response to a uniform electrical input. Temporal characterization was then conducted by actuating all nodes simultaneously at 150 V while sweeping the input frequency from 2 to 800 Hz. Three separately fabricated devices were tested.

The lateral force response, shown in Fig. 3 (B and C), was relatively constant at 12.43 ± 0.75 mN for low frequencies (<100 Hz) and then decreased to a lower steady-state level of 8.21 ± 0.79 mN at higher frequencies. In contrast, the bulk finger vibrations’ response peaked near 77 Hz (Fig. 3, E and F). All vibrations could be perceived by users in the haptic demonstration (see the “Haptic demonstrations” section), and the vibration magnitudes were also compared to published human vibrotactile frequency thresholds (*8*). The recorded vibration amplitudes exceeded perceptual thresholds across this full range, indicating a perceptible bandwidth of at least 500 Hz. While psychophysical threshold data beyond 500 Hz are limited, the trend suggests that VoxeLite remains effective well into the higher frequency range.

### Characterizing spatial resolution

As previously demonstrated in (*29*), pure lateral motions generated by frictional forces create large couplings resulting from the lateral stiffness of the skin, resulting in a spatial resolution of only 5 mm. As such, a key motivation for using nodes that generate normal indentations is to avoid coupling effects and enable spatially independent actuation. To evaluate whether independent node motion could be achieved and to quantify the resulting normal indentations, we developed a transparent biomimetic finger sensor (see Materials and Methods).

The sensor consisted of three layers: a rigid acrylic base simulating bone, a thick soft intermediate layer representing bulk finger tissue, and a thin, stiffer outer layer mimicking the stratum corneum. These layers were chosen as they captured the essential differences in normal and lateral stiffness across finger layers. Prior work by Srinivasan (*40*) also showed that a three-layer model was sufficient for predicting surface deflection in a finger.

The sensor used a sensing principle similar to Johnson and Adelson (*41*). The outermost layer was coated with a reflective membrane to map indentation profiles. When an object is pressed against the sensor, the reflective coating deforms to match the object’s profile (fig. S6, A and B), and darkened regions are proportional to the indentation depth. A high-speed camera [1280 by 960 pixels, 296 frames per second (fps)] recorded the resulting deformation. The sensor was mounted on the tribometer in the same configuration used for human finger testing, and VoxeLite was applied identically. The same normal force (0.5 N) and swiping speed (20 mm/s) were also used. Unlike human calibration, sensor alignment was performed until all nodes showed uniform responses to identical signals. The sensor can also be used to observe either through the top or from the front. When viewed from the front, the sensor was back-lit to increase visualization contrast, and when viewed from the top, the sensor was lit in parallel to the camera (fig. S6, C and D).

**Fig. 4.**
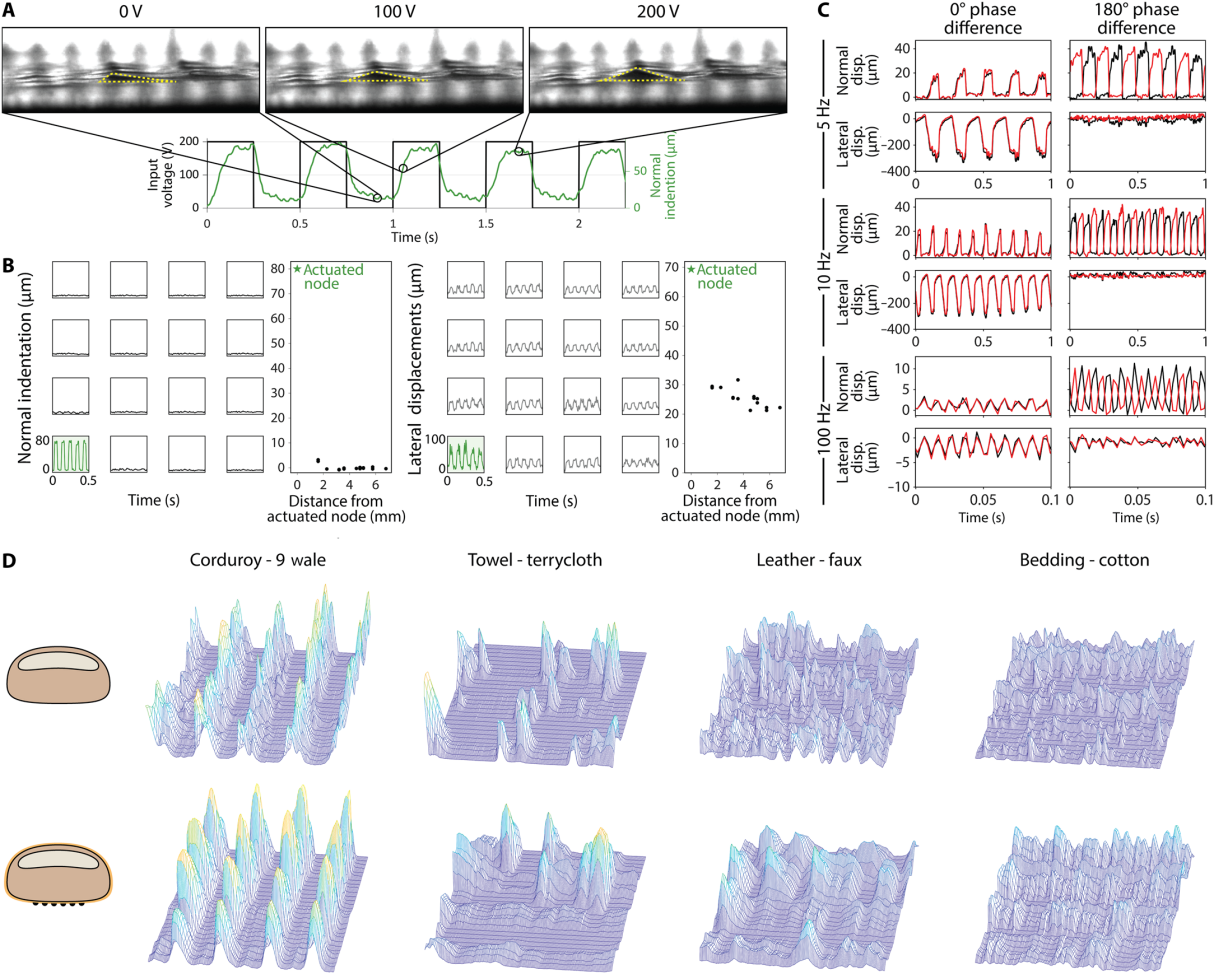
Spatial and transparency characteristics of the haptic device. (**A**) Normal indentations were recorded with a transparent biomimetic finger sensor when a node was driven with a 2-Hz, 200-V signal. (**B**) Normal and lateral displacements of each node in an array when only the bottom left-most node is actuated. (**C**) Mean normal and lateral displacements of an array actuated in a checkerboard pattern. Red lines are the means of one group actuated with the same signal, and black lines are the means of the second group actuated with the same voltage and frequency but with a given phase delay from the red group. (**D**) SEPs depicting the spatial and temporal variations of a fingerpad experience when touching four different types of textures. The SEPs are compared when the finger is either bare or wearing VoxeLite.

To evaluate independent motion, we actuated only the bottom-left node at 10 Hz and 150 V while keeping all other nodes passive. From the front view, the shape of indentation can be seen in Fig. 4A, and the maximum normal indentation generated was 80.56 ± 3.91 μm. A baseline indentation of 11.67 ± 3.73 μm was observed because of static friction, yielding an average dynamic peak-to-peak displacement (∆*x*) of 68.9 μm postslip.

From the top view, lateral and normal displacements were extracted for all nodes (Fig. 3B and movie S1). In the normal direction, no coupling was observed. Nonactuated nodes showed a minimal displacement average ∆*x* of 0.14 ± 1.17 μm. However, a coupling effect was observed in the lateral direction, consistent with (*29*). The actuated node had an average lateral ∆*x* of 69.32 μm, while the furthest passive node (6.79 mm away) had an average ∆*x* of 22.23 μm.

We further explored phase-based actuation patterns across the array (Fig. 3C). All nodes were driven at 150 V in a checkerboard pattern with phase delays between neighboring nodes of 0°, 45°, 90°, 135°, and 180°, and frequencies of 5, 10, and 100 Hz. To achieve a higher temporal resolution, the camera spatial resolution was reduced to 1280 by 720 pixels, and the frame rate was increased to 389.6 fps.

At the 0° phase difference, all nodes moved synchronously in the normal and lateral directions. The average ∆*x* in the lateral direction decreased as the frequency increased, changing from 309.1 μm (5 Hz) to 4.1 μm (100 Hz). Normal indentations followed the same trend, decreasing from 20.1 μm (5 Hz) to 2.79 μm (100 Hz). At the 180° phase difference, different behaviors were seen in the normal and lateral directions. In the normal direction, all nodes had similar ∆*x* and maintained the imposed phase offset, confirming minimal mechanical coupling. In the lateral direction, motions were significantly dampened, where the average ∆*x* values were only 38.2 μm at 5 Hz and 2.47 μm at 100 Hz. This effect is consistent with coupling effects arising from lateral skin stiffness.

### Characterizing haptic transparency

We also evaluated VoxeLite’s ability to transmit real tactile information to the fingertip by measuring spatial and temporal variations during passive exploration. The transparent sensor was mounted onto the tribometer and used in its top-view configuration. Two test conditions were compared: (i) a bare finger condition, where nothing was worn on the sensor, and (ii) a device-on condition, where VoxeLite was attached to the sensor. Eight fabrics from four distinct texture classes were selected: 9-wale and 14-wale corduroy, faux and full-grain leather, microfiber and terrycloth towels, and polyester and cotton bedding. These samples were chosen to span a wide range of tactile properties, including differences in macrotexture, microtexture, compliance, and friction (fig. S7).

Sensor outputs were processed similarly to that described by Connor and Johnson (*42*). For each fabric, mechanical spatial event plots (SEPs) were generated from eight target locations distributed across the fingerpad surface (Fig. 4D and fig. S8). Each SEP was convolved with 1D and 2D Gabor filters to extract temporal and spatial variations, respectively (see Materials and Methods for full processing details).

Unlike the SEPs in (*42*), which were derived from neural recordings, our SEPs were constructed from skin displacement measurements. This approach yielded qualitatively similar patterns, as tactile afferent firing rates can be approximated by the mechanical stresses, or proportionally the displacements, experienced by the skin (*43*). We therefore treated displacement as a proxy for neural activity. However, we note that this simplified model does not capture the dynamic propagation of deformation across the skin, which will result in additional neural responses.

Under the bare finger condition, SEPs from different locations on the fingerpad surface exhibited minimal variability, consistent with uniform tactile transmission across the surface. In contrast, when VoxeLite was worn, the tactile stimuli became spatially discretized by the array structure. Locations aligned with the edges of the nodes produced amplified signals that closely matched the bare finger response, while locations between nodes exhibited attenuated responses. This introduced variability across the finger surface. However, the overall spatial and temporal variations remained well correlated with that of the bare finger condition (fig. S9 and movies S3 to S10).

Overall, VoxeLite faithfully transmitted the spatial and temporal patterns associated with each fabric. With the exception of terrycloth (which has a complex macrostructure because of pulled threads), the resulting spatial and temporal features closely matched those recorded under the bare finger condition. Although VoxeLite had slightly amplified signals, the quantitative trends across fabric types remained consistent, demonstrating the device’s ability to preserve key tactile cues during passive texture exploration.

### Haptic demonstrations

To demonstrate the breadth of haptic information that VoxeLite can convey in both its active and passive states, we conducted three user experiments. The first two evaluated the array’s capabilities in the active mode, where participants identified either hapticons or virtual textural patterns. The third experiment tested VoxeLite in its passive mode, where participants performed a matching task involving real-world fabrics.

Experiments one and two were conducted using the tribometer. Tactile signals were only delivered when moving to the right, the normal force was maintained at 0.2 N, and the finger’s incidence angle was adjusted individually to ensure full contact between the array and the grounded surface. Participants used their dominant index fingers and wore headphones playing pink noise to disguise any apparatus sound.

The third experiment was conducted without active actuation and off the tribometer. Participants performed a matching task using the previously tested eight fabrics. Each participant completed the task under three conditions: with a bare finger, with a latex sheath (0.5 mm thick) on the finger, and with VoxeLite.

Fifteen participants (aged 28.1 ± 4 years, five females) were recruited for the first experiment, and a second group of 15 participants (aged 28.6 ± 5.8 years, five females) took part in the third experiment. All study procedures were approved by the Northwestern University Institutional Review Board. Participants provided informed consent and were compensated for their time.

#### Small-scaled hapticons

**Fig. 5.**
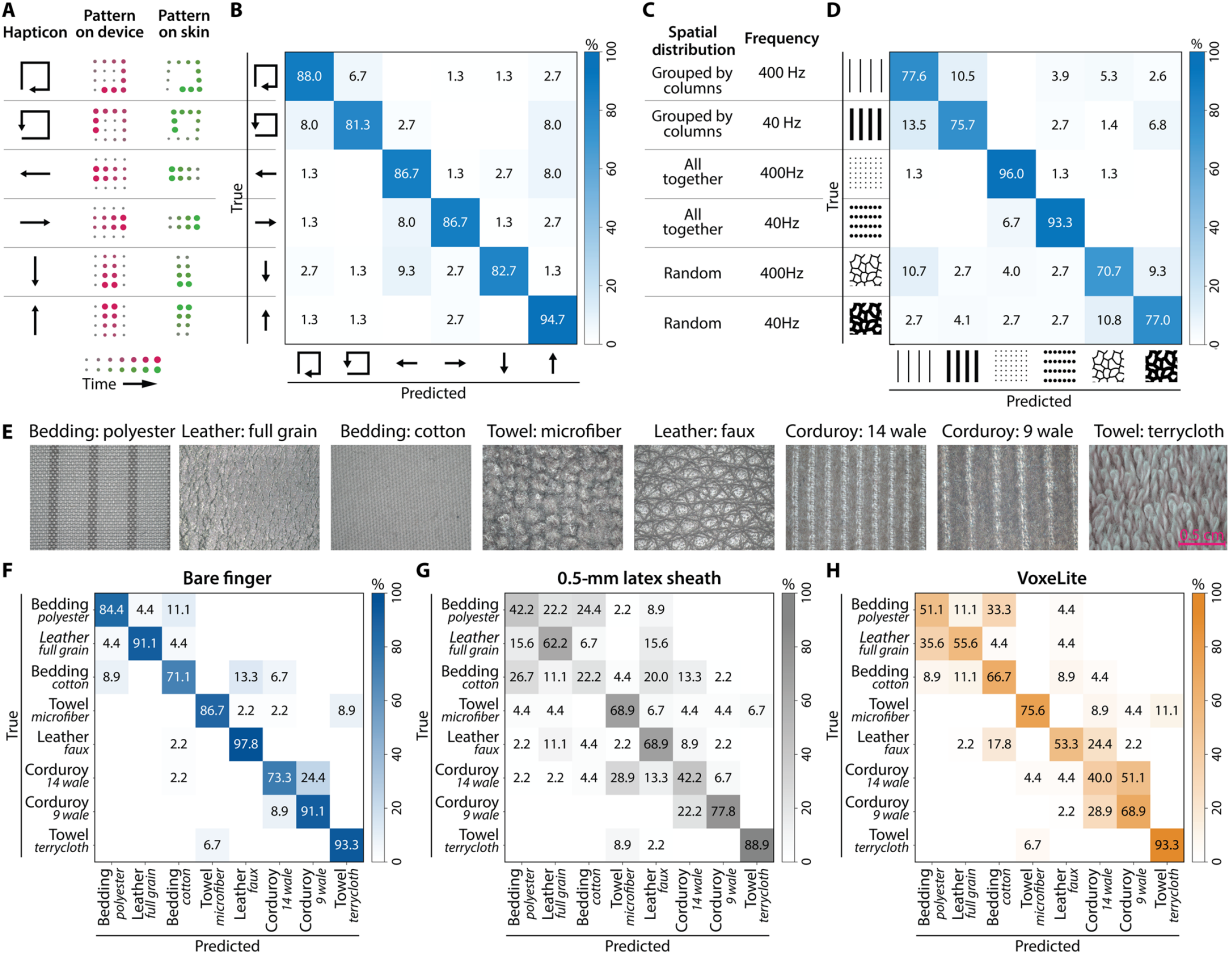
Results of user studies assessing the effectiveness of our haptic device in presenting different tactile information. (**A**) Spatiotemporal patterns used to create different hapticons. (**B**) Confusion matrix results for tactile stimuli presenting small-scaled hapticons within the fingerpad. (**C**) Spatial distributions and frequencies used to generate different texture renderings. (**D**) Confusion matrix results when users are presented with texture-based tactile stimuli. (**E**) Micrographs of the eight real textures used in the transparency test and characterizations. (**F** to **H**) Confusion matrix results testing the device transparency in three conditions: bare finger, finger covered with a 0.5-mm latex sheath, and finger covered with VoxeLite.

To demonstrate VoxeLite’s ability to render fine spatial information, we conducted a discrimination task using a 5 by 5–mm patch of the fingerpad. Six hapticons (up, down, left, right, clockwise, and counterclockwise) (*44*) were encoded using spatiotemporal patterns generated by sequentially actuated nodes. Stimuli were presented only when the finger moved toward the right at a speed of 2 mm/s for 3.75 s. Nothing was played during the first 0.25 s to ensure that the finger had fully slipped before stimulus onset. The slow speed was used to minimize the distortion of the perceived direction (Fig. 5A).

For each direction, the nodes were actuated sequentially for 50 ms with no overlap. After completing a full actuation sequence, a pause of 300 ms was introduced to provide perceptual anchors at the beginning and end of each stimulus.

Before the start of the test, participants completed a training phase in which they were presented with all six directions and could freely replay without a time limit. Most participants completed training after two to three replays per direction. During the test, participants completed 30 randomly ordered trials, each consisting of five trials per direction. Participants could replay each stimulus as many times as needed before submitting a response. All participants reported perceiving the indentation patterns and were able to identify directional motion with an average accuracy of 86.7 ± 4.7% (Fig. 5B). This demonstrated that VoxeLite can deliver interpretable, high-resolution spatial cues.

#### Texture patterns

VoxeLite’s high spatiotemporal resolution also enables it to convey distinct texture sensations. To demonstrate this, we compared six virtual texture patterns that varied along two dimensions: (i) spatial distribution of the actuated nodes and (ii) signal frequency.

Three spatial distributions were tested: (i) column-wise, where each vertical column of nodes was actuated sequentially at the same frequency; (ii) synchronous, where all nodes were actuated simultaneously; and (iii) randomized, where each node had the same actuation frequency but was assigned a unique phase offset. Each spatial distribution was tested at two actuation frequencies, 40 and 400 Hz (Fig. 5C).

Similar to the direction task, stimuli were only presented when moving to the right. Here, the movement speed was increased to 20 mm/s over a 2-s duration, with no signal presented during the first 0.25 s. Participants performed the same training protocol used in the direction task. All participants reported clear percepts of the tactile patterns and were able to distinguish the various textures. The average accuracy was 81.7 ± 10.4% (Fig. 5D). The highest accuracies were observed for synchronous actuation, likely due to its perceptually distinct lack of spatial variation. Under this condition, nodes moving in sync also produced the strongest signal at 40 Hz (96%) and the weakest signal at 400 Hz (93.3%).

#### Evaluating haptic transparency

Our final investigation aimed to assess whether VoxeLite permitted natural surface exploration and transmitted real tactile information without perceptual or mechanical interference. Using the same eight fabric samples from the haptics transparency characterization, participants performed matching tasks with their right index finger under three conditions: (i) bare finger, (ii) latex sheath, and (iii) VoxeLite. Under each condition, participants used their right index finger to explore a sample fabric and attempted to match it to one of eight fabrics presented to their left index finger. Participants verbally reported their match and were instructed to use only their index fingers throughout the task. The left index finger remained bare, and they could not see their hands or the fabrics. The order of the three conditions was randomized for each participant, and participants provided a confidence rating after each condition.

Participants adopted similar exploratory strategies across all conditions, typically scanning from left to right with both hands. Participants were given opportunities to rest during and between trials, but no participant reported fatigue or movement restrictions when using either the latex sheath or VoxeLite. This suggests that the added mass and form factor of VoxeLite did not hinder natural exploration.

Under the bare finger condition, participants achieved an overall discrimination accuracy of 86.1%. In comparison, the latex sheath and VoxeLite conditions yielded accuracies of 59.2 and 63.1%, respectively. While the overall accuracy with VoxeLite was comparable to that of the latex sheath, errors made with VoxeLite more closely correlated with those from the bare finger condition (*r* = 0.765 for device and *r* = 0.495 for latex) (Fig. 5, F to H). Participants using the latex sheath also reported a general dampening of tactile feedback, resulting in difficulty distinguishing between rough and smooth textures. In contrast, VoxeLite preserved broader category-level distinctions: Classification errors remained confined within “rough” and “smooth” texture groups.

We hypothesize that the inability to discriminate finer texture details was due to the limited spatial resolution of the device used in this experiment. The tested version featured a 6-by-6 array with a center-to-center node spacing of 1.6 mm. This is larger than the known spatial acuity of the human fingertip. In addition, the nodes were arranged in a regular square grid. This uniform layout may have further reduced the fidelity of subtle or irregular texture features by imposing a structured sampling pattern that does not align with the natural variability of real surfaces.

## DISCUSSION

This work presents a class of wearable haptic interfaces capable of delivering high-resolution spatiotemporal tactile feedback in a lightweight, skin-conformal form factor. Leveraging scalable microfabrication techniques, VoxeLite achieves actuator densities up to 110 nodes/cm^2^ and a perceptual bandwidth of 800 Hz. Each node is individually addressable, enabling precise control over localized mechanical indentations and allowing the generation of complex, temporally modulated tactile patterns at the fingertip. The fabrication method is also readily extensible to various density arrays and alternative configurations, supporting broader investigations into spatial encoding for virtual touch. Owing to its thin, flexible design, VoxeLite can also transmit real-world tactile information to the skin. This haptic transparency enables seamless transitions between natural and virtual touch, allowing users to interact with physical objects, such as handling tools, without the need to remove the device. Psychophysical studies validated the system’s ability to produce perceptually rich directional cues and texture-like patterns. By combining high-density spatiotemporal actuation with wearability, VoxeLite addresses key limitations in current haptic displays and opens opportunities for immersive virtual and augmented reality, interactive robotics, and advanced human-machine interfaces. In practice, these capabilities could support accessibility applications (e.g., helping blind users trace contours for navigation), enable intuitive tactile identification and localization of buttons on touch-enabled devices, and enhance interactions in virtual reality/augmented reality environments (Fig. 2C).

However, several challenges remain before VoxeLite can be fully translated into large-scale, deployable systems. Our fabrication process prioritized actuator density, accessibility with limited industrial-level equipment, and high manufacturing yield (100% for interfaces with fewer than 36 actuators, decreasing as the actuator count increases). Scaling to multifinger coverage or more complex form factors will require optimized routing architectures and alternative fabrication strategies to reduce defects introduced at each fabrication step. This work also focused primarily on mechanical optimization of the fingertip interface, with the current electronics supporting only tethered operation. Achieving untethered, system-level integration will require further development of compact and wireless electronics, including strategies for efficient multiplexing and signal delivery within a wearable form factor.

Beyond engineering challenges, understanding how users perceive and adapt to dense spatiotemporal stimulation during long-term use remains an important area for future work. Individual differences in skin mechanics may require personalized calibration protocols, while variations in temperature, perspiration, and sustained tactile input can influence both user comfort and perceptual performance. Maintaining consistent adhesion between the device and skin after extended use or under high perspiration levels may also pose additional challenges.

Last, there remains limited knowledge of the optimal node densities and spatial configurations needed to maximize tactile realism and communication fidelity. Future work will focus on advancing personalized calibration methods, exploring different strategies to further enhance tactile realism and communication fidelity, and developing alternative attachment methods to ensure long-term comfort and reliable skin contact.

## MATERIALS AND METHODS

### Fabrication of tactile array

Natural latex (HX-200, Holden’s Latex) was used as the base material, and a CNT dispersion (Tuball latex, OCSiAl) was added to increase the latex’s conductivity. Urethane rubber (Reoflex 40, Smooth-On) was used as the filler material for each node as it was not a cure inhibitor of latex. Conductive traces were patterned using flexible silver ink (118-43, Creative Materials). Our fabrication process is illustrated in fig. S1.

All devices are made with an acrylic mold machined with hemispheres of a wanted diameter, spacing, and layout. Conductive latex was prepared by mixing natural latex, CNT suspension, and deionized (DI) water in a 1:1:2 ratio. DI water was added to reduce the solution’s viscosity for easier pipetting. The conductive latex mixture was pipetted into each hemisphere cavity and filled completely. The mold was then cured in an oven at 80°C for 5 min, leaving only a thin film of conductive latex coating the bottom of each hemisphere. A 1:2 mixture of DI water and latex was then airbrushed over the mold and cured at 80°C for another 5 min. The pipetting and airbrushing process was repeated two more times. Once completed, the flat portion of the mold that did not contain the hemispheres was dip coated in latex to form a thicker layer. This was followed by a 10-min cure at 80°C. Once fully cured, the inner surface of each hemisphere was coated with silver ink. The remaining electrode traces were created via stencil laser cutting (ProtoLaser R4, LPKF Laser & Electronics) from a 7-μm sheet of Mylar film. Internode traces were 0.11 mm wide, while all remaining traces were 0.18 mm wide. The mold was left to cure at 80°C for 10 min after which the hemispheres were filled with urethane rubber and cured at 80°C for 4 hours. The entire mold was dip coated again in latex and cured for another 10 min. The encapsulated device was demolded using talc powder to prevent adhesion between surfaces. Flat flexible cables (PSR1635-16, Parlex USA LLC) were attached using pressure-sensitive conductive tape (electrically conductive adhesive transfer tape 9703, 3M). Overall, the latex between the nodes was ~0.1 mm thick and did not exhibit any perceptible coupling. Latex in other regions was 0.15 mm thick to enhance mechanical durability during handling and connection.

To permanently reduce surface friction, the latex component of VoxeLite was chlorinated twice. Each chlorination cycle involved soaking the latex in a 1000:30:5 mixture of DI water, bleach, and muriatic acid for 3 min. VoxeLite was then rinsed in DI water for 10 min to remove residual chemicals. After rinsing, it was air dried for at least 5 hours to ensure complete evaporation of moisture before use.

### Fabrication of anodized aluminum

The anodized aluminum countersurface was fabricated from mirror-finished aluminum plates. Before anodization, the plates were cleaned with soap and rinsed thoroughly. They were then submerged in an electrolytic bath composed of a 10:1 solution of DI water to sulfuric acid. A second aluminum plate was used as the cathode.

The solution was held in a double-chamber setup, with the outer chamber circulating water to maintain a stable temperature during the anodization process. A current density of 0.013 A/cm^2^ was applied for 30 min. After anodization, the plate was rinsed with a sodium bicarbonate solution to neutralize any remaining acid. Last, the part was immersed in a sealing bath of boiling DI water for 10 min.

### Fabrication of the biomimetic finger sensor

The biomimetic finger consisted of three layers: a 1.6-mm-thick acrylic layer (representing bone); 0.1-MPa, 1.5-cm-thick silicone inner layer (representing the dermis); and a 1-MPa, 0.4-mm-thick silicone outer layer (representing the stratum corneum). Aside from the acrylic, all layers were made from an optically transparent silicone (XP-565, Sterling Supply Inc.), and the modulus was controlled by varying the ratio of parts A and B.

The sensor was cast in a 3D-printed mold that had a similar curvature to a human finger. To fabricate it, a thin layer of 10:1 mixture of XP-565 was first applied to the bottom of the mold and cured for 1 hour at 60°C. A 20:1 mixture of XP-565 was then poured on top to fill the remainder of the mold. This was cured for 2 hours at 60°C. After curing, the bilayer silicone was demolded. Silver powder was applied to the stiffer outer layer and encapsulated with a thin coating of Ecoflex 00-10. This layer also serves as an adhesive layer to attach VoxeLite and was left to cure at room temperature for 30 min. A piece of rigid acrylic was adhered to the softer inner silicone layer, and a 3D-printed bracket was mounted above the acrylic to allow attachment to the tribometer.

### Optimizing electrostatic forces generation of nodes

The electrostatic forces that can be generated by VoxeLite are governed by the standard equation for force between an air-filled parallel plate capacitor

$$F=\frac{A\varepsilon_{0}\varepsilon_{g}}{2}{\left(\frac{V_{g}}{d_{g}}\right)}^{2}$$ (1)

where *A* is the area of contact, ε_0_ is the permittivity of free space, ε_g_ is the relative gap permittivity, *V*_*g*_ is the gap between the surfaces, and *d*_*g*_ is the gap between the plates.

From our node stackup, *V*_*g*_ is not the applied voltage but an attenuated version that is reduced by the resistance from the dielectric layer of the countersurface and the resistance of the outermost layer of the node. A simple model of *V_g_* is thus

$$V_{g}=V_{t}\left(\frac{R_{g}}{R_{g}+R_{d}+R_{n}}\right)$$ (2)

where *V*_*t*_ is the total voltage, *R*_*d*_ is the bulk resistance of the dielectric layer, *R*_*g*_ is the gap resistance, and *R*_*n*_ is the bulk resistance of the outermost layer of the node (conductive latex). Combining Eqs. 1 and 2 gives

$$F=\frac{A\varepsilon_{0}\varepsilon_{g}}{2}{\left(\frac{V_{t}}{d_{g}}*\frac{R_{g}}{R_{g}+R_{d}+R_{n}}\right)}^{2}$$ (3)

For a given voltage, generating the highest displacement required us to maximize *F*. In our design, we can do so by increasing *A* and minimizing *R*_*d*_ and *R*_*n*_. To reduce the design space, the dielectric layer on the anodized aluminum was kept constant during manufacturing. *A* and *R*_*n*_ could instead be respectively optimized by changing the stiffness of the filler rubber in the node and the conductivity of the outermost node layer.

To evaluate the effect of *R*_*n*_, the modulus of the node was kept constant at Shore 40A, while the concentration of CNTs in the outermost conductive latex layer was varied. CNTs (0.83, 1.65, 2.48, and 4.1 wt %) were tested. Separately, the effect of changing *A* was evaluated using constant 2.48 wt % CNTs and by changing the stiffness of the nodes to Shore 20, 40, and 60A. All tests were performed on the tribometer with all nodes in a given array actuated synchronous at 100 Hz. Tests were performed from 60 to 200 V, in increments of 10 V (fig. S2).

To verify the effect of Shore hardness on *A*, frustrated total internal reflection imaging under a constant normal load was used. Using the tribometer, the countersurface was switched to a transparent glass and was lit from the side. A camera was placed under the glass to capture the change in contact area (fig. S3A).

The area of contact was further varied by changing the normal preload between the nodes and the countersurface. All tested devices were fabricated with 2.48 wt % CNTs and Shore 40A hardness. The normal preload was varied from 0.2 to 1 N, in increments of 0.2 N (fig. S4). All nodes were actuated synchronously at 160 V and frequencies of 50, 100, and 200 Hz. Changes in the area of contact under different preloads were also quantified using the above frustrated total internal reflection imaging method (fig. S3B).

### Node performance limits

For all versions of VoxeLite, the nodes shared the same hemispherical geometry. When used against a smooth flat surface, normal indentations can only be generated by actively tilting the hemispherical nodes. As such, the maximum normal displacement *y* achievable on a smooth flat surface is

y=r∗sinθ (4)

where *r* is the radius of the node, and θ is the tilt angle.

Because of the passive nature of the device, lateral resistance from high-friction or uneven surfaces can induce pretilt, limiting the normal displacements a node can generate. Any preexisting dc indentation, from a dc actuation voltage, inherent surface friction, or microgeometries, further reduces the available range of indentation during active actuation, thus decreasing the effective active tactile output. When using smooth surfaces, as in all tests reported here, actuation remains well above the perceptual threshold.

### Multichannel control system

To generate a multichannel high-voltage controller, each node was connected to a high-voltage converter module made from an Operational Amplifier (LT1636, Analog Devices Inc. Instruments), two transistors (ZTX958, Diode Incorporated, and FJP5027OTU, onsemi), and passive components (resistors, capacitors, and diodes). The module received signals from a digital-to-analog converter (MCP4922, Microchip Technology) that was connected to a PIC32MX795F512H microcontroller. The high-voltage source was an MPM12-500P that was current limited to 3 mA (fig. S10).

### Evaluating haptic transparency through SEPs

For each texture, the soft sensor scanned a surface while a camera recorded the deformations at 1280 by 960–pixel resolution and 296.45 fps. For each video, eight target locations were selected: four at the outer edge of two nodes, two at the centers of the same two nodes, and two between adjacent nodes. At each target location, a column of 29 receptive fields was defined, oriented perpendicular to the scanning direction. Each receptive field corresponded to a 0.2 by 0.2–mm area [the same as that used in (*42*)], and the pixel values within each field were averaged to produce a single height value.

This process was repeated across all fields over 650 frames (2.21 s). For each target location, the time series of height values was plotted as rows of event markers, and the rows were stacked on top of each other to form a raster plot, similar to how SEPs were created in (*42*). Overall, each scan across a texture resulted in eight mechanical SEPs corresponding to the eight target locations.

Each SEP was then convolved with 1D and 2D Gabor filters to quantify the temporal and spatial variations, respectively. The Gabor filters and their corresponding parameters were based on the equation optimized in (*42*). No further parameter optimization was performed.

The 1D Gabor filter used was

$$f\left(t\right)=\text{sin}\left(\frac{2\pi t}{\lambda }+\varphi \right)*\text{exp}\left(-\frac{t^{2}}{2\sigma^{2}}\right)$$ (5)

where *t* is the time, λ = 64 ms is the temporal period of the sinusoid, φ is the phase of the sinusoid relative to *t* = 0, and σ = 51 is the standard deviation of the Gaussian distribution. All results were averages across (φ = 0, π/2, π, 3π/4). The resolution of the filter was the same as the camera’s frame rate, and the same firing rate of 4 ms was adopted from (*42*).

The 2D Gabor filter used was

$$f\left(t\right)=\text{sin}\left[\frac{2\pi \left(x*\text{sinθ}-y*\text{cosθ}\right)}{\lambda }+\varphi \right]*\;\text{exp}\left(-\frac{x^{2}+y^{2}}{2\sigma^{2}}\right)$$ (6)

where *x* and *y* are the spatial positions, θ is the orientation of the spatial sinusoidal component of the filter, λ = 2.8 mm is the spatial period of the sinusoid component of the filter, and σ = 1.12 mm is the standard deviation of the 2D Gaussian. All results were averages across φ = 0, π/2, π, and 3π/4 and θ = 0°, 30°, 60°, 90°, 120°, and 150°. The filter resolution was 0.2 mm.

For a given texture, the temporal and spatial variations calculated from each SEP were averaged to obtain a single representative value of temporal and spatial variation. This process was repeated when the sensor was bare, covered with a 0.5-mm latex sheath, and covered with VoxeLite.

## Supplementary Materials

### The PDF file includes:

Figs. S1 to S10 Table S1 Legends for movies S1 to S10 References
