# Supplementary material for: Toward human-resolution haptics: A high-bandwidth, high-density, wearable tactile display
Source: Sci Adv. 2025 Nov 19;11(47):eadz5937. doi: 10.1126/sciadv.adz5937 (PMC12629187; doi:10.1126/sciadv.adz5937)
Supplement: Supplementary file 1 — Figs. S1 to S10 Table S1 Legends for movies S1 to S10 References [file sciadv.adz5937_sm.pdf]

Science Advances
AAAS

Supplementary Materials for

Toward human-resolution haptics: A high-bandwidth, high-density, wearable tactile display

Sylvia Tan *et al.*

Corresponding author: Sylvia Tan, sylviatan@u.northwestern.edu

Sci. Adv. **11**, eadz5937 (2025)
DOI: 10.1126/sciadv.adz5937

The PDF file includes:

Figs. S1 to S10
Table S1
Legends for movies S1 to S10
References

Other Supplementary Material for this manuscript includes the following:

Movies S1 to S10

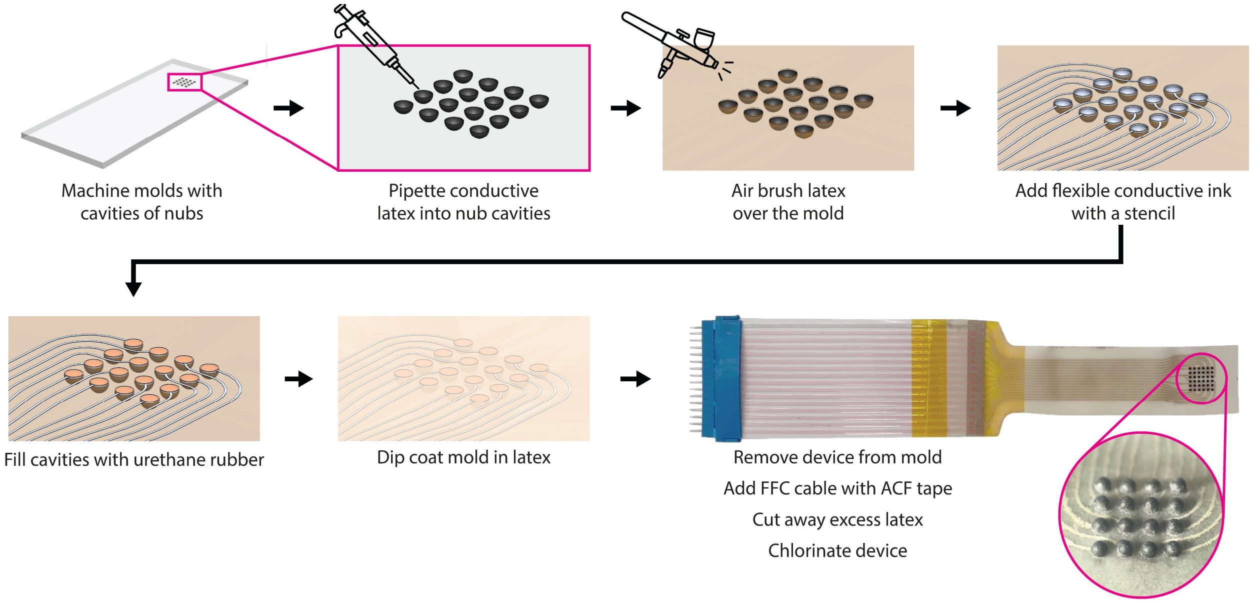

Fig. S1. Full fabrication process of making a device. Different array sizes are made by changing the mold and stencil for routing the connections.

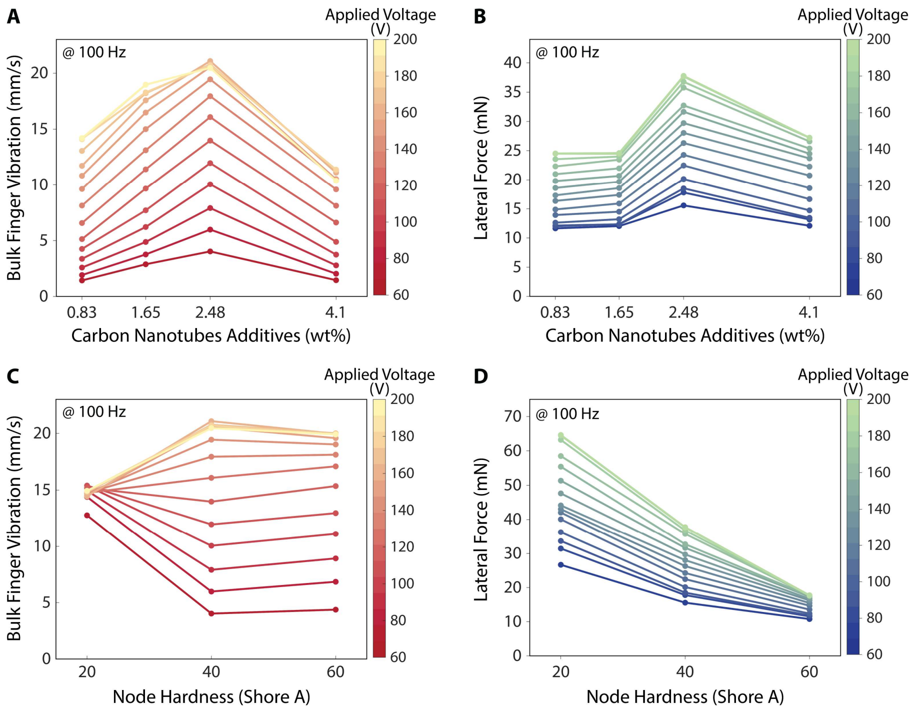

Fig. S2. Optimization of electrical and mechanical properties of the device. All characterization was done with all nodes actuated simultaneously at 100 Hz, at voltages from 60 - 200 V, in increments of 10 V. **(A-B)** Effect of changing the amount of carbon nanotube additives to the bulk finger vibration and lateral forces that can be generated. All devices had a Shore 40 A modulus. **(C-D)** Effect of changing the node modulus on the bulk finger vibration and lateral forces. All devices used a 2.48 wt % carbon nanotube additive.

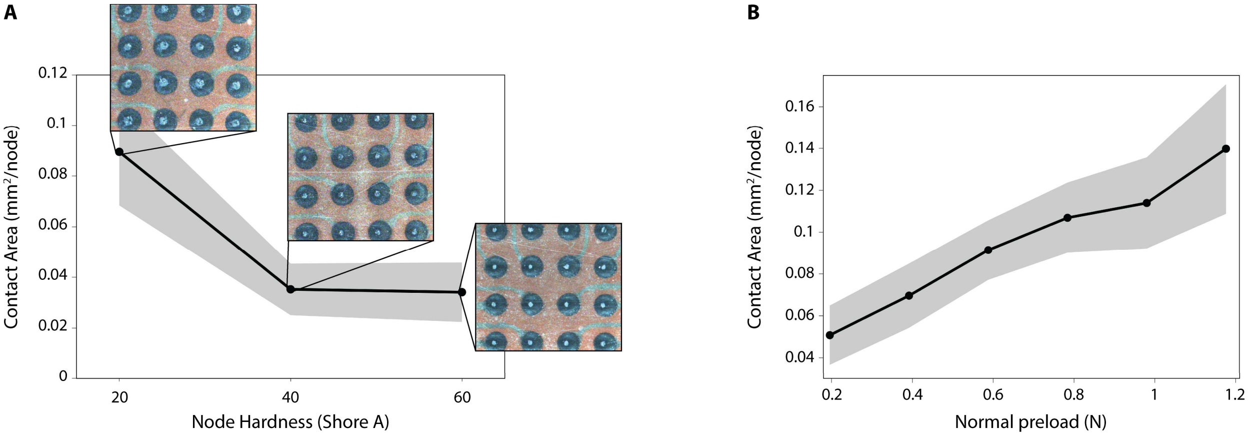

Fig. S3. Contact Area change in response to a change in (A) node modulus with a constant normal preload of 0.5 N and (B) normal preload with a constant hardness of shore 40A. The nodes are pressed onto a glass surface, and light is emitted through the side of the glass. Using the principle of Frustrated total internal reflection (FTIR), the areas in contact with the glass light up. The bright circles at the center of each node are the portions of the nodes that are in contact.

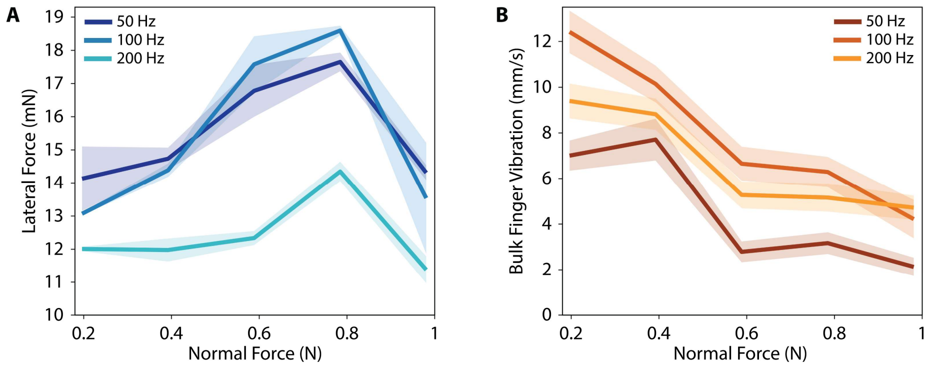

Fig. S4. Optimization of normal preload for tests with tribometer. All characterization was performed with all nodes actuated simultaneously at 160 V, across three frequencies, and with normal preloads ranging from of 0.2 to 1.0 N, in increments of 0.2 N. All devices used a 2.48 wt % carbon nanotube additive and shore 40A hardness. (A) Effect of varying preload on the generated lateral forces, and (B) effect of varying preload on the bulk finger vibrations.

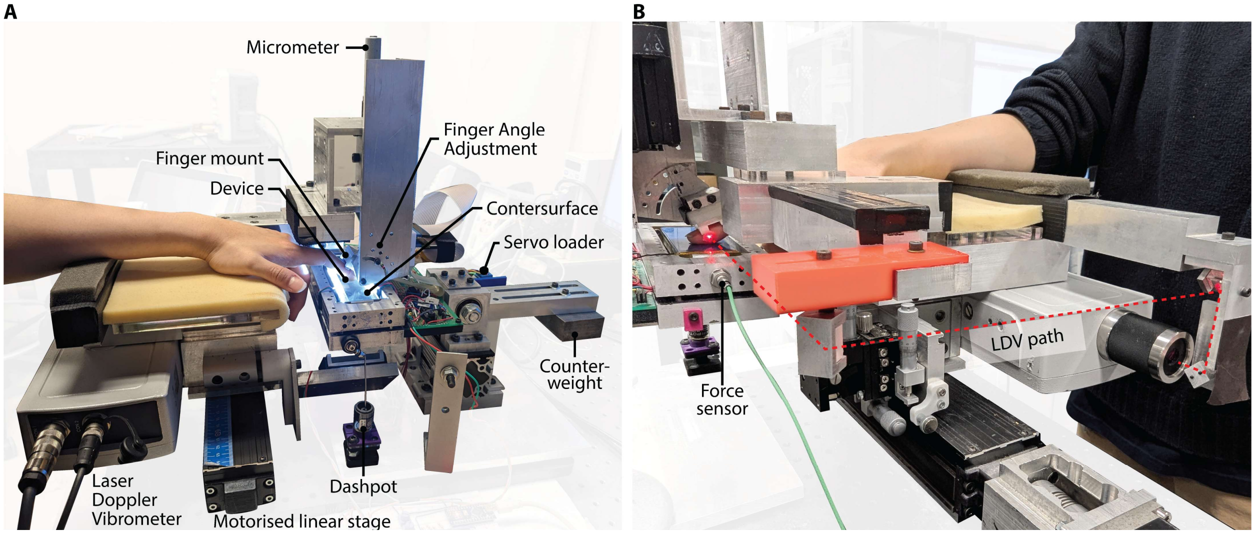

Fig. S5. Custom tribometer setup used for characterization and running perceptual tests. (A) Right side view of the tribometer. The motions of the user's hands and index finger are controlled by the setup, the forces acting on the finger are also controlled and measured. **(B)** Left side view of the setup. The red line indicates the pathway the Laser Doppler Vibrometer takes to measure the vibration at the side of the finger. The lateral force sensor is attached to the side of the countersurface and measures the lateral forces.

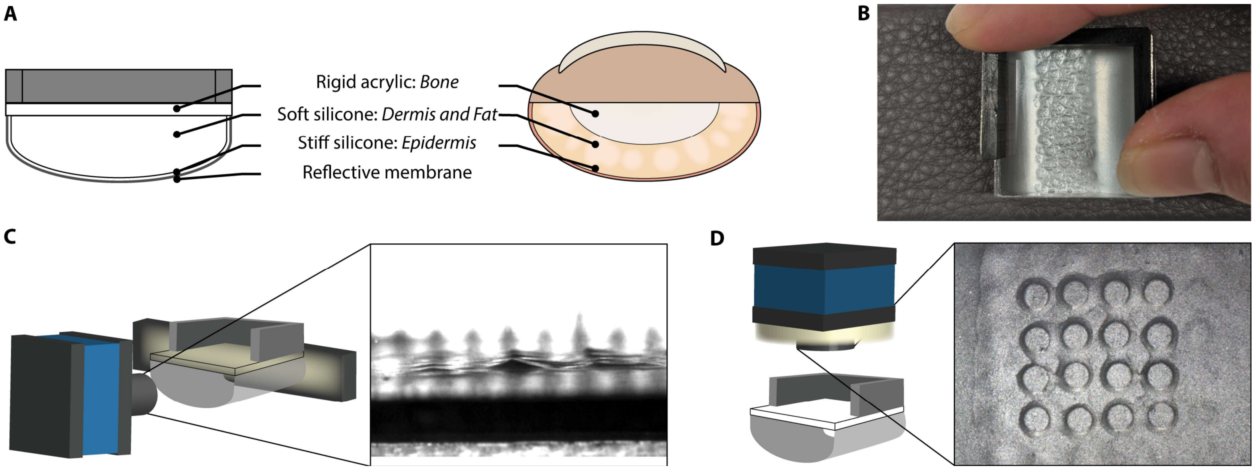

Fig. S6. Biomimetic transparent sensor developed to evaluate within-finger variations. (A) Layers in the sensor that mimic layers in the finger. (B) Distortion is seen in the sensor when it is pushed against faux leather. The image is captured with a high-speed camera. (C) The sensor used in a front-facing configuration to capture normal indentation into the sensor. (D) The sensor in the top-facing configuration, normal indentation is into the plane where darker region indicates higher indentations.

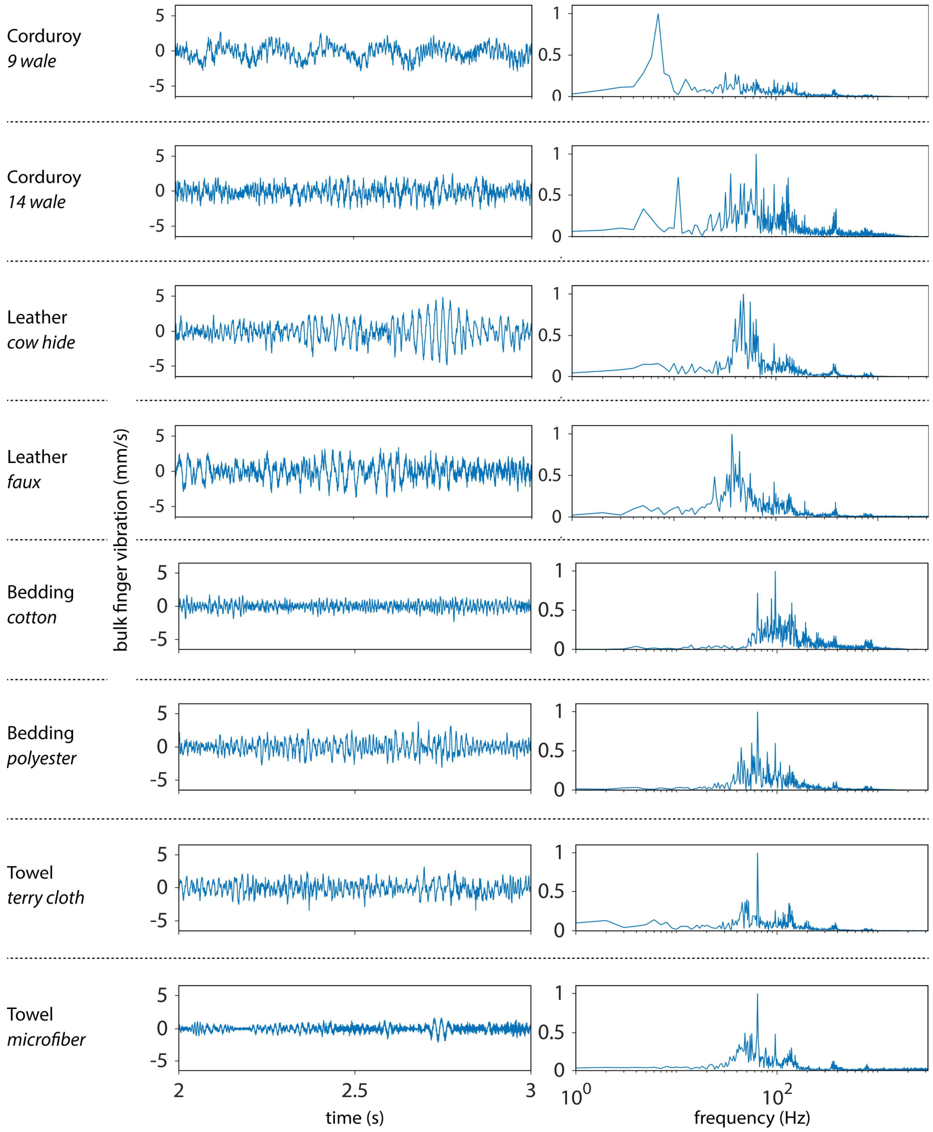

Fig. S7. Bulk Finger Variations of a finger swiping over the 9 real tested textures.

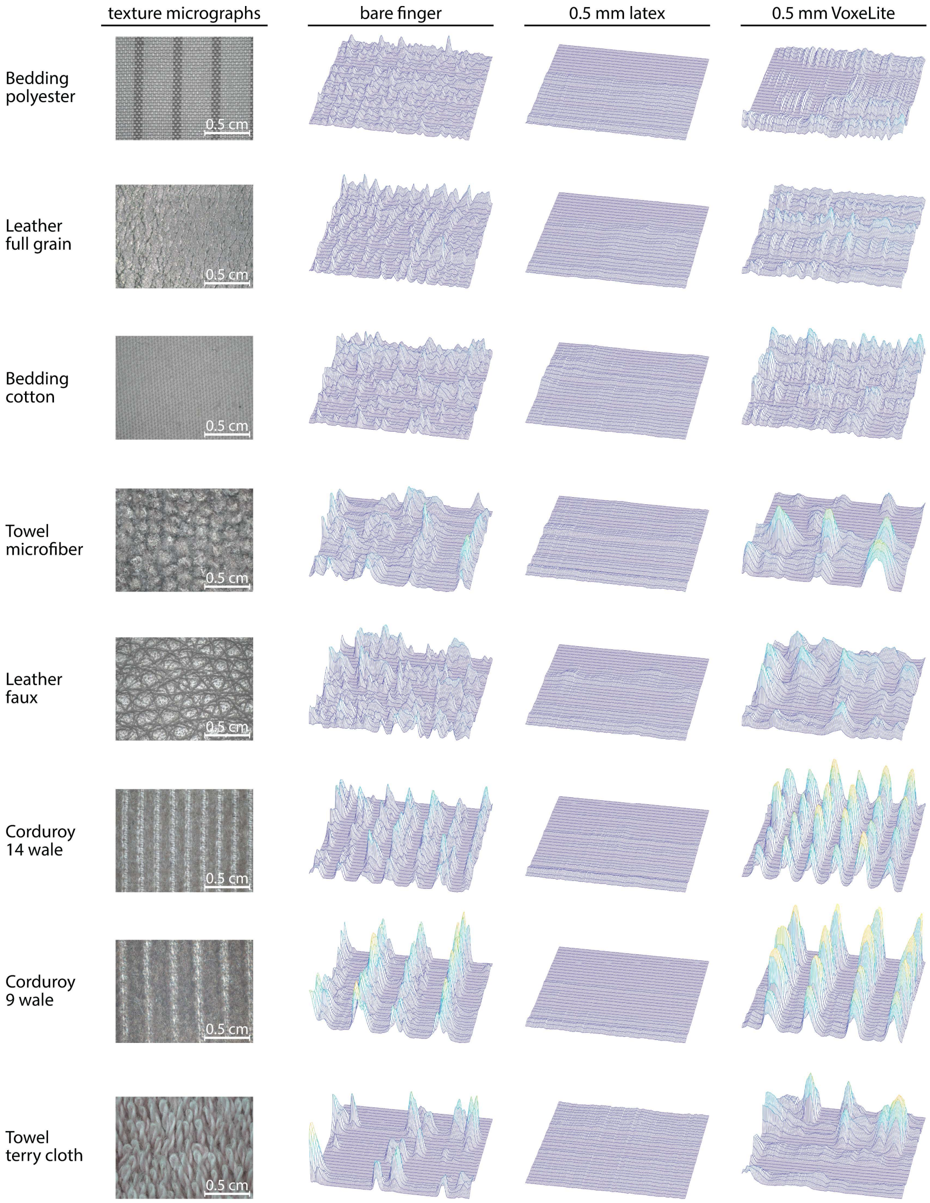

Fig. S8. Comparisons of SEPs obtained when the sensor is swiped over 8 textures, and in three conditions: (1) bare finger, (2) covered with a 0.5 mm latex sheath, and (3) covered with VoxeLite. Surface microstructures of each texture are all included to provide a reference to the SEPs that are generated in each condition.

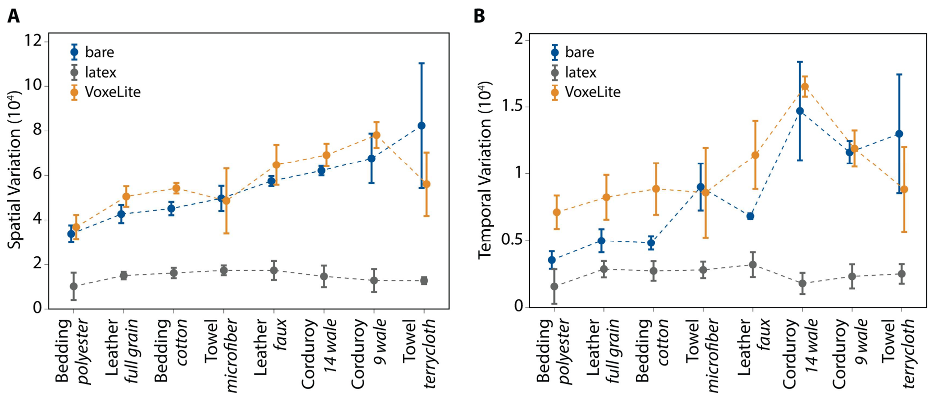

Fig. S9. The (A) spatial and (B) temporal variations for eight fabrics tested in three conditions: (1) bare finger, (2) latex sheath, and (3) VoxeLite. The fabrics are ordered according to the spatial variations in the bare finger condition.

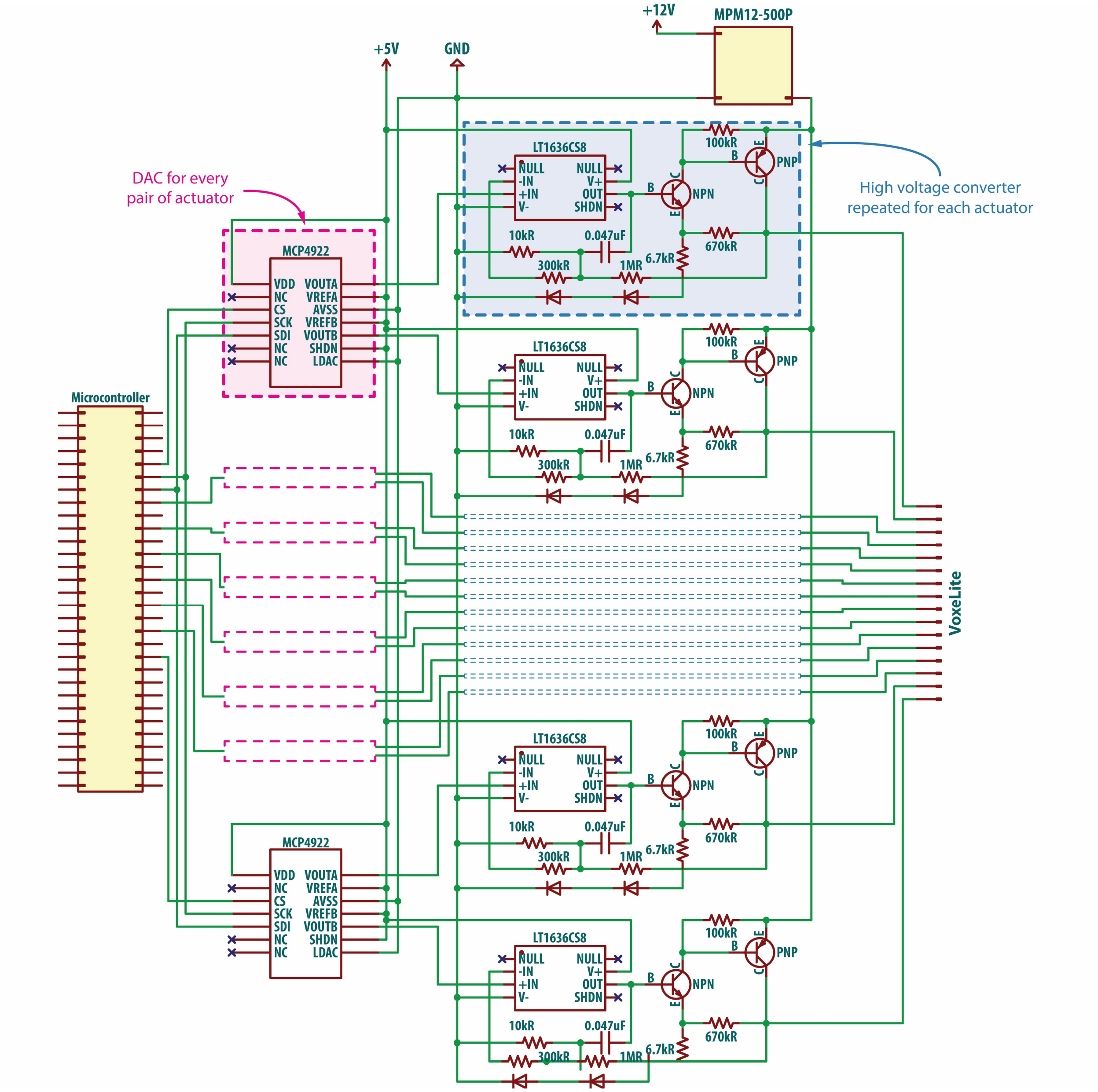

Fig. S10. Example of the basic electrical setup to drive 16 nodes individually. Each node has its own high voltage amplifier that converts a 0 - 5 V input signal to a 0 - 500 V output signal. For safety, the high voltage source has a maximum current capacity of 3 mA. When tested on the tribometer, VoxeLite and the tribometer are all controlled and programmed through the same microcontroller to ensure motions are in sync.

Table S1. Comparisons of human tactile resolution to existing wearable and tabletop tactile display.

Ref	Form factor	Weight (mg/actuator)	Actuation type	Spatial Resolution (actuator/cm^2)	Temporal bandwidth (Hz)
(46,47)	Mechanoreceptor	-	RA I	140	190
(46,47)	Mechanoreceptor	-	RA II	21	930
(46,47)	Mechanoreceptor	-	SA I	69	100
VoxeLite: 8x8	Wearable	4.6875	Electroadhesion	110.80	800
VoxeLite: 6x6	Wearable	5.16667	Electroadhesion	44.44	800
VoxeLite: 4x4	Wearable	22.5625	Electroadhesion	19.75	800
22	Wearable	2	DEA	12.99	150
23	Wearable	2600	DEA	0.21	400
24	Wearable	1300	DEA	1.06	500
25	Wearable	35.6	DEA	3.52	500
26	Wearable	-	Multilayer DEA	0.21	500
29	Wearable	62.5	Electroadhesion	18.52	600
27	Wearable	-	Electrohydraulic	20.00	320
28	Wearable	250	Electrohydraulic	16.00	700
17	Wearable	800	Motors (LRA)	44.44	140
37	Wearable	5200	Motors (LRA)	0.15[a]	200
38	Wearable	100000	Motors (LRA)	0.15[a]	235
31	Wearable	-	Pneumatic	0.32	100
32	Wearable	531.25	Pneumatic	23.80	100
33	Wearable	-	Electrotactile	76.00	<1000[b]
34	Wearable	-	Electrotactile	0.15[a]	<1000[b]
35	Wearable	-	Electrotactile	2.86	<1000[b]
10	tabletop	-	Ultrasonic	0.15[a]	<1000[b]
11	tabletop	-	Vibrotactile + Electrostatic	0.15[a]	-
14	tabletop	-	Electroadhesion	0.15[a]	<1000[b]
12	tabletop	-	Piezoelectric	0.15[a]	500
18	tabletop	-	Piezoelectric	31.80	250
19	tabletop	1000	Piezoelectric	60.00	-
15	tabletop	-	Dielectric fluid	14.11	200
16	tabletop	4804.69	Pneumatic	66.67	50
20	tabletop	-	Voicecoil	20.90	80
21	tabletop	-	Custom voicecoil	26.81	450

[a] Displays consist of 1 actuator for the entire finger pad. An average fingerpad area of 6.6 cm was used during calculation [45]

[b] No frequency responses were obtained, but the actuation method suggests high frequency bandwidths

Movie S1.
Video of VoxeLite attached to the transparent biomimetic sensor and a single node is actuated at 150V and 10Hz.

Movie S2.
Side profile video of VoxeLite attached to a real finger and all nodes are actuated at 125V.

Movie S3.
Videos of the transparent biomimetic sensor swiping over 9 wale corduroy in 3 conditions (1. Bare finger, 2. Latex sheath, and 3. VoxeLite)

Movie S4.
Videos of the transparent biomimetic sensor swiping over 14 wale corduroy in 3 conditions (1. Bare finger, 2. Latex sheath, and 3. VoxeLite)

Movie S5.
Videos of the transparent biomimetic sensor swiping over faux leather in 3 conditions (1. Bare finger, 2. Latex sheath, and 3. VoxeLite)

Movie S6.
Videos of the transparent biomimetic sensor swiping over full grain leather in 3 conditions (1. Bare finger, 2. Latex sheath, and 3. VoxeLite)

Movie S7.
Videos of the transparent biomimetic sensor swiping over polyester bedding in 3 conditions (1. Bare finger, 2. Latex sheath, and 3. VoxeLite)

Movie S8.
Videos of the transparent biomimetic sensor swiping over cotton bedding in 3 conditions (1. Bare finger, 2. Latex sheath, and 3. VoxeLite)

Movie S9.
Videos of the transparent biomimetic sensor swiping over microfiber towel in 3 conditions (1. Bare finger, 2. Latex sheath, and 3. VoxeLite)

Movie S10.
Videos of the transparent biomimetic sensor swiping over terrycloth towel in 3 conditions (1. Bare finger, 2. Latex sheath, and 3. VoxeLite)

REFERENCES AND NOTES

1. M. Schadt, Milestone in the history of field-effect liquid crystal displays and materials. *Jpn. J. Appl. Phys.* **48**, 03B001 (2009).

2. W. Zhang, P. N. Samarasinghe, H. Chen, T. D. Abhayapala, Surround by sound: A review of spatial audio recording and reproduction. *Appl. Sci.* **7**, 532 (2017).

3. T. Field, *Touch* (The MIT Press, 2001).

4. A. Montagu, *Touching: The Human Significance of the Skin* (Columbia Univ. Press, 1971).

5. J. B. F. van Erp, A. Toet, Social touch in human–computer interaction. *Front. Digit. Hum.* **2**, 2 (2015).

6. K. O. Johnson, J. R. Phillips, Tactile spatial resolution. i. Two-point discrimination, gap detection, grating resolution, and letter recognition. *J. Neurophysiol.* **46**, 1177–1192 (1981).

7. R. W. V. Boven, K. O. Johnson, The limit of tactile spatial resolution in humans. *Neurology* **44**, 2361–2361 (1994).

8. S. J. Bolanowski Jr., G. A. Gescheider, R. T. Verrillo, C. M. Checkosky, Four channels mediate the mechanical aspects of touch. *The J. Acoust. Soc. Am.* **84**, 1680–1694 (1988).

9. L. R. Manfredi, H. P. Saal, K. J. Brown, M. C. Zielinski, J. F. Dammann III, V. S. Polashock, S. J. Bensmaia, Natural scenes in tactile texture. *J. Neurophysiol.* **111**, 1792–1802 (2014).

10. L. Winfield, J. Glassmire, J. E. Colgate, M. Peshkin, "T-pad: Tactile pattern display through variable friction reduction," in *Second Joint EuroHaptics Conference and Symposium on Haptic Interfaces for Virtual Environment and Teleoperator Systems (WHC'07)* (IEEE, 2007), pp. 421–426.

11. K. Otake, H. Hasegawa, S. Okamoto, Y. Yamada, "Virtual roughness textures via a surface tactile texture display using vibrotactile and electrostatic friction stimuli: Improved realism," in *2020 13th International Conference on Human System Interaction (HSI)* (IEEE, 2020), pp. 147–152.

12. M. Wiertlewski, J. Lozada, V. Hayward, The spatial spectrum of tangential skin displacement can encode tactual texture. *IEEE Trans. Robot.* **27**, 461–472 (2011).

13. C. Basdogan, F. Giraud, V. Levesque, S. Choi, A review of surface haptics: Enabling tactile effects on touch surfaces. *IEEE Trans. Haptics* **13**, 450–470 (2020).

14. R. V. Grigorii, R. L. Klatzky, J. E. Colgate, Data-driven playback of natural tactile texture via broadband friction modulation. *IEEE Trans. Haptics* **15**, 429–440 (2022).

15. A. K. Han, S. Ji, D. Wang, M. R. Cutkosky, Haptic surface display based on miniature dielectric fluid transducers. *IEEE Robot. Autom. Lett.* **5**, 4021–4027 (2020).

16. Y. Ujitoko, T. Taniguchi, S. Sakurai, K. Hirota, Development of finger-mounted high-density pin-array haptic display. *IEEE Access* **8**, 145107–145114 (2020).

17. Q. Wu, J. Li, H. Seifi, K. Hornbæk, *Vipins*: Combining a pin array and vibrotactile actuators to render complex shapes and textures. *Int. J. Hum. Comput. Stud.* **197**, 103464 (2025).

18. Y. Ikei, K. Wakamatsu, S. Fukuda, Vibratory tactile display of image-based textures. *IEEE Comput. Graph. Appl.* **17**, 53–61 (1997).

19. Q. Wang, V. Hayward, Biomechanically optimized distributed tactile transducer based on lateral skin deformation. *Int. J. Rob. Res.* **29**, 323–335 (2010).

20. R. V. Grigorii, J. E. Colgate, R. L. Klatzky, The spatial profile of skin indentation shapes tactile perception across stimulus frequencies. *Sci. Rep.* **12**, 13185 (2022).

21. Y. Massalim, D. Faux, V. Hayward, Distributed tactile display with dual array design. *IEEE Trans. Haptics* **16**, 334–338 (2023).

22. I. M. Koo, K. Jung, J. C. Koo, J. Nam, Y. K. Lee, H. R. Choi, Development of soft-actuator-based wearable tactile display. *IEEE Trans. Robot.* **24**, 549–558 (2008).

23. J. H. Youn, H. Mun, K. U. Kyung, A wearable soft tactile actuator with high output force for fingertip interaction. *IEEE Access* **9**, 30206–30215 (2021).

24. X. Ji, X. Liu, V. Cacucciolo, Y. Civet, A. E. Haitami, S. Cantin, Y. Perriard, H. Shea, Untethered feel-through haptics using 18-μm thick dielectric elastomer actuators. *Adv. Funct. Mater.* **31**, 2006639 (2021).

25. J. H. Youn, S. Y. Jang, I. Hwang, Q. Pei, S. Yun, K. Kyung, Skin-attached haptic patch for versatile and augmented tactile interaction. *Sci. Adv.* **11**, eadt4839 (2025).

26. S. Mun, S. Yun, S. Nam, S. K. Park, S. Park, B. J. Park, Electro-active polymer based soft tactile interface for wearable devices. *IEEE Trans. Haptics* **11**, 15–21 (2018).

27. V. Shen, T. Rae-Grant, J. Mullenbach, C. Harrison, C. Shultz, "Fluid reality: High-resolution, untethered haptic gloves using electroosmotic pump arrays," in *UIST '23: Proceedings of the 36th Annual ACM Symposium on User Interface Software and Technology* (Association for Computing Machinery, 2023).

28. Purnendu, J. Hartcher-O'Brien, V. Mehta, N. Colonnese, A. Gupta, C. J. Bruns, "Fingertip wearable high-resolution electrohydraulic interface for multimodal haptics," in *2023 IEEE World Haptics Conference (WHC)* (IEEE, 2023), pp. 299–305.

29. S. Tan, R. L. Klatzky, M. A. Peshkin, J. Edward Colgate, Pixelite: A thin and wearable high bandwidth electroadhesive haptic array. *IEEE Trans. Haptics* **16**, 555–560 (2023).

30. R. Hinchet, V. Vechev, H. Shea, O. Hilliges, "DextrES: Wearable haptic feedback for grasping in VR via a thin form-factor electrostatic brake," in *UIST '18: Proceedings of the 31st Annual ACM Symposium on User Interface Software and Technology* (Association for Computing Machinery, 2018), pp. 901–912.

31. H. A. Sonar, A. P. Gerratt, S. P. Lacour, J. Paik, Closed-loop haptic feedback control using a self-sensing soft pneumatic actuator skin. *Soft Robot.* **7**, 22–29 (2020).

32. N. Morita, A. Ichijo, M. Konyo, H. Kato, K. Sase, H. Nagano, S. Tadokoro, Wearable high-resolution haptic display using suction stimuli to represent cutaneous contact information on finger pad. *IEEE Trans. Haptics* **16**, 687–694 (2023).

33. W. Lin, W. Lin, D. Zhang, W. W. Lee, X. Li, Y. Hong, Q. Pan, R. Zhang, G. Peng, H. Z. Tan, Z. Zhang, L. Wei, Z. Yang, Super-resolution wearable electrotactile rendering system. *Sci. Adv.* **8**, eabp8738 (2022).

34. Y. Tanaka, A. Shen, A. Kong, P. Lopes, "Full-hand electro-tactile feedback without obstructing palmar side of hand," in *CHI '23: Proceedings of the 2023 CHI Conference on Human Factors in Computing Systems* (Association for Computing Machinery, 2023).

35. S. Y. Teng, A. Gupta, P. Lopes, "Haptic permeability: Adding holes to tactile devices improves dexterity," in *CHI '24: Proceedings of the 2024 CHI Conference on Human Factors in Computing Systems* (Association for Computing Machinery, 2024).

36. J.-H. Kim, A. Vázquez-Guardado, H. Luan, J.-T. Kim, D. S. Yang, H. Zhang, J.-K. Chang, S. Yoo, C. Park, Y. Wei, Z. Christiansen, S. Kim, R. Avila, J. U. Kim, Y. J. Lee, H.-S. Shin, M. Zhou, S. W. Jeon, J. M. Baek, Y. Lee, S. Y. Kim, J. Lim, M. Park, H. Jeong, S. M. Won, R. Chen, Y. Huang, Y. H. Jung, J.-Y. Yoo, J. A. Rogers, A wirelessly programmable, skin-integrated thermo-haptic stimulator system for virtual reality. *Proc. Natl. Acad. Sci. U.S.A.* **121**, e2404007121 (2024).

37. P. Preechayasomboon, E. Rombokas, Haplets: Finger-worn wireless and low-encumbrance vibrotactile haptic feedback for virtual and augmented reality. *Front. Virtual Real.* **2**, 738613 (2021).

38. H. Kim, M. Kim, W. Lee, "Hapthimble: A wearable haptic device towards usable virtual touch screen," in *CHI '16: Proceedings of the 2016 CHI Conference on Human Factors in Computing Systems* (Association for Computing Machinery, 2016).

39. J. Vaicekauskaite, P. Mazurek, S. Vudayagiri, A. L. Skov, Mapping the mechanical and electrical properties of commercial silicone elastomer formulations for stretchable transducers. *J. Mater. Chem. C* **8**, 1273-1279 (2020).

40. M. Srinivasan, Surface deflection of primate fingertip under line load. *J. Biomech.* **22**, 343–349 (1989).

41. M. K. Johnson, E. H. Adelson, Retrographic sensing for the measurement of surface texture and shape, in 2009 IEEE Conference on Computer Vision and Pattern Recognition (2009). https://doi.org/10.1109/cvpr.2009.5206534.

42. C. Connor, K. Johnson, Neural coding of tactile texture: comparison of spatial and temporal mechanisms for roughness perception. *J. Neurosci.* **12**, 3414–3426 (1992).

43. H. P. Saal, B. P. Delhaye, B. C. Rayhaun, S. J. Bensmaia, Simulating tactile signals from the whole hand with millisecond precision. *Proc. Natl. Acad. Sci. U.S.A.* **114**, E5693–E5702 (2017).

44. M. Enriquez, K. MacLean, "The hapticon editor: A tool in support of haptic communication research," in *11th Symposium on Haptic Interfaces for Virtual Environment and Teleoperator Systems, 2003. HAPTICS 2003. Proceedings* (IEEE, 2003), pp. 356–362.

45. B. M. Dzidek, M. J. Adams, J. W. Andrews, Z. Zhang, S. A. Johnson, Contact mechanics of the human finger pad under compressive loads. *J. R. Soc. Interface* **14**, 20160935 (2017).

46. R. S. Johansson, A. B. Vallbo, Tactile sensibility in the human hand: relative and absolute densities of four types of mechanoreceptive units in glabrous skin. *J. Physiol.* **286**, 283–300 (1979).

47. M. Park, B. G. Bok, J. H. Ahn, M. S. Kim, Recent advances in tactile sensing technology. *Micromachines* **9**, 321 (2018).
